# Aspects of acceptance: building a shared conceptual understanding

**DOI:** 10.3389/fpsyg.2024.1423976

**Published:** 2024-06-21

**Authors:** Michael D. Mrazek, Bailey R. Dow, Justine Richelle, Alexander M. Pasch, Nathan Godderis, Talia A. Pamensky, Bryce A. Rutila, Alissa J. Mrazek

**Affiliations:** ^1^School of Public Health, The University of Texas Health Science Center at Houston (UTHealth), Austin, TX, United States; ^2^Michael & Susan Dell Center for Healthy Living, Austin, TX, United States; ^3^School Psychology, Department of Psychology, University of South Carolina, Columbia, SC, United States; ^4^The Applied Psychology Laboratory, Department of Psychology, The University of Texas at Austin, Austin, TX, United States

**Keywords:** acceptance, acknowledgement, allowing, non-judgment, non-attachment

## Abstract

Many contemplatives, scientists, and clinicians have pointed to the value of responding to life’s difficulties by accepting experiences as they are. A growing body of research also suggests that acceptance contributes to effective coping with adversity, reduced stress, and improved emotional well-being. Yet within the scientific literature, there is little consensus on what acceptance means or how it should be measured. This makes it nearly impossible to synthesize empirical work on acceptance into a cohesive scientific understanding. Our goal in this paper is to clarify four facets of acceptance that are commonly referenced in research: acknowledging, allowing, non-judging, and non-attachment. We do not propose a specific definition of acceptance or even a set of privileged facets that must be included in future frameworks. We instead offer a vocabulary to facilitate productive communication among researchers that will, in turn, enable a more definitive scientific understanding of this important construct to emerge. After defining and explaining these aspects of acceptance, we further clarify these constructs in two ways. First, we illustrate how the four aspects are dissociable from one another. Second, we analyze their correspondence to related constructs from Acceptance and Commitment Therapy (ACT). Finally, we provide a concept worksheet that scholars can utilize to precisely operationalize acceptance in their own work.

## 1 Introduction

As wondrous as life can be, adversity and emotional difficulties are inevitable parts of the human experience. For millennia, religious and contemplative traditions have emphasized the importance of acceptance in dealing effectively with these challenges (Williams and Lynn, 2010; Shah, 2021). In everyday situations, acceptance can help us come to terms with both internal and external challenges, which can lead to greater resilience and emotional well-being. Hundreds of scientific studies have explored this idea, with many concluding that acceptance carries numerous psychological benefits (Ranzijn and Luszcz, 1999; Roemer and Orsillo, 2002; Hayes et al., 2004a,b; Ford et al., 2018; Zhao et al., 2020; Klussman et al., 2022).

But what exactly is acceptance? It is unsurprising that the word acceptance has a variety of meanings in common usage. Yet among scientists conducting research on acceptance, one would expect a shared understanding of what precisely this word means. Unfortunately, that is far from the case. As this paper demonstrates, there are a multitude of definitions and interpretations of acceptance. This, in turn, makes it difficult to discern when scientists are theorizing, measuring, and manipulating the same construct.

To understand the risks entailed in continuing research on acceptance without greater conceptual clarity, the related field of mindfulness can serve as a cautionary tale. The number of published studies into mindfulness has grown exponentially (Baminiwatta and Solangaarachchi, 2021), with many thousands of papers published in the last decade. In theory, this amount of research should have produced a body of conclusive findings regarding the effects of mindfulness training, as well as key moderators and mechanisms of action. Yet meta-analyses and systematic reviews regularly lament the lack of consistency in findings, often citing the absence of consensus in construct specificity and operational definitions as a root cause (Bishop et al., 2004; Mooneyham et al., 2016; Van Dam et al., 2018; Anālayo, 2019; Phan-Le et al., 2022). Indeed, there are numerous validated self-report instruments for measuring mindfulness in different ways, as well as dozens of unique mindfulness training programs, each with its own perspective on what mindfulness conveys (Nilsson and Kazemi, 2016; Grossman, 2019).

In the foreseeable future, scientists are unlikely to adopt universally shared definitions of complex constructs like mindfulness or acceptance. Yet for continued research to produce definitive findings that build into a coherent scientific understanding, it is essential that researchers at least communicate their respective definitions and frameworks in ways that allow for synthesis across studies.

Our goal in this paper is to specify and clarify several of the facets that are commonly referenced in research on acceptance. We do not propose a specific definition of acceptance or even a collection of privileged facets that must be included in future frameworks of acceptance. Instead, we hope to provide a vocabulary that can help researchers communicate productively. We first review four aspects of acceptance that are frequently referenced in the scientific literature: acknowledging, allowing, non-judging, and non-attachment. Although these four aspects do not necessarily arise in any particular sequence, we present them in the order they most commonly unfold. We then explain how these facets can be distinguished from one another. Finally, we illustrate the aspects of acceptance by exploring how they are incorporated into Acceptance and Commitment Therapy—an evidence-based approach to psychotherapy that weaves together aspects of mindfulness, acceptance, and related constructs. We conclude by providing a concept worksheet that researchers can utilize in their future work to clearly communicate their chosen definition and framework.

## 2 Aspect one: acknowledging

Often the first step of accepting something is acknowledging that it exists. The human mind has a fascinating capacity to deny the existence of things that should be easily recognizable. When initially faced with tragedy, people so often struggle to confront their new reality that denial has historically been considered the first stage of grief (Kübler-Ross, 1981). Similarly, traumatic experiences can sometimes be repressed for years before resurfacing as recovered memories (Williams, 1995; McNally, 2005). Furthermore, some people manage to deny their least redeeming qualities and behaviors not only to others but even to themselves (Blaine and Crocker, 1993).

Given the human mind’s ability to deny the existence of an experience, acknowledgement is a non-trivial facet of acceptance. Acknowledgement is sometimes referred to as “being aware of” or “having knowledge of” something (Govier, 2003; Grossman, 2019). In the grief literature, it is defined as an “initial recognition” or in layman’s terms “reality sinking in” (Love, 2007; Blandin and Pepin, 2017). Yet perhaps confusingly, the word is also sometimes used to describe not only the initial recognition but also additional steps that come after. In the disability literature, for example, acknowledgement has been defined as the recognition of the reality of one’s situation combined with the gradual integration of that reality into one’s self-concept (Livneh and Antonak, 1990). In service of avoiding terms with complex, multi-dimensional definitions, here we define acknowledgement as simply the recognition of something’s existence. In this sense, the phenomenology of acknowledgment is more conceptual than sensory or emotional.

The effective measurement of acknowledgment is deeply challenged by the fact that people cannot accurately report on what they do not experience. Yet there may still be some merit to self-report instruments that assess conscious efforts at denial or repression. Perhaps the most relevant self-report measure of acknowledgment is the Brief COPE scale. It includes four items, three of which directly address acknowledgement as we define it: “I’ve been saying to myself this is not real” (reverse coded), “I’ve been refusing to believe that it has happened” (reverse coded), and “I’ve been accepting the reality of the fact that it has happened” (Carver, 1997).

The fact that individuals could self-report on how much they have acknowledged something suggests that there is a middle ground between complete denial and complete acknowledgement. Partial acknowledgement means that there is some recognition of something’s existence but at the same time some continued denial (Govier, 2003). For example, a recently bereaved widow might at some level acknowledge that her spouse has died while nevertheless appearing to think, act, and live as though nothing has changed. Despite some recognition of the new reality, there is also some continued denial, which can arise through deliberate effort or unconscious processes.

## 3 Aspect two: allowing

Another aspect of acceptance is the willingness to not only acknowledge something but to fully experience it. Even when someone conceptually acknowledges an aversive part of their life, they may understandably want to avoid the ongoing experience of it. Many of us try to soothe discomfort, suppress unpleasant thoughts, and push away uncomfortable feelings. Indeed, it is often adaptive to avoid or change aversive experiences—covering one’s ears in the presence of a deafening sound is an appropriate and protective response.

Yet one can also develop an excessive pattern of avoiding or suppressing experiences that becomes counterproductive to the point of self-sabotage (Brereton and McGlinchey, 2020; Hayes-Skelton and Eustis, 2020; Cybulska et al., 2022). This pattern is sometimes referred to as experiential avoidance, which has been defined as the “attempt to avoid or escape private events, even when the attempt to do so may cause psychological harm” (Hayes et al., 1996).

Accordingly, many conceptualizations of acceptance emphasize the importance of allowing experiences to exist without attempting to suppress or avoid them (Hayes et al., 2012). The act of suppression has been previously distinguished from the act of repression (Hsu, 1949). In the Freudian sense, repression occurs when thoughts and feelings are buried so deeply that they are hidden from conscious awareness and cannot be readily accessed. A repressed thought is therefore an unacknowledged thought. In contrast, suppression attempts to push conscious thoughts and emotions out of awareness. So whereas we construe acknowledgment as recognizing that something exists rather than repressing it, allowing would be permitting that thing to exist in your experience over time rather than suppressing it. People can allow something to exist in their experience in at least two ways. First, they can actively direct their attention to an experience and thereby bring it to the forefront of their mind. Alternatively, they could simply disengage any effort to actively suppress an experience and thereby permit it to arise unimpeded.

Allowing is featured prominently in many mindfulness frameworks, where it is sometimes framed as a willingness to remain in contact with one’s experience. Allowing has also been described as “being experientially open to the reality of the present moment” and as “an attitude of openness and receptivity” (Roemer and Orsillo, 2002; Bishop et al., 2004). Although allowing is widely considered an aspect of acceptance, there is less consensus about the details. The word allowing is sometimes used interchangeably with a range of similar verbs such as non-interfering, tolerating, enduring, embracing, welcoming, and warmly regarding (Cordova, 2001; Lindsay and Creswell, 2019). Yet some disagreement exists as to whether allowing entails a neutral orientation to any given experience or a positive and welcoming orientation (Williams and Lynn, 2010). For example, some have contended that acceptance should not be equated with tolerance because they view acceptance as the active embrace of an experience rather than the mere tolerance of it (Hayes et al., 2004a,b).

Another important point of divergence is whether allowing applies similarly to “inner” and “outer” experiences. The scientific literatures on mindfulness and experiential avoidance often describe allowing as a way of relating to “inner” or private experiences like bodily sensations, thoughts, and emotions. However, some researchers have also conceived of allowing in the context of “outer” experiences, such as whether we try to control other people or let them be as they are (Ilie, 2021).

## 4 Aspect three: non-judging

A third aspect of acceptance involves how we interpret an experience. Like a sports commentator analyzing a game, our minds provide a running commentary that shapes how we experience our unfolding lives. For many people, a significant portion of this commentary consists of evaluations (also commonly referred to as judgments) that appraise the quality, importance, or value of something. These evaluations can have an especially powerful effect on how we perceive events and, by extension, how we feel about them (Ellsworth and Scherer, 2003). It is therefore unsurprising that many conceptualizations of acceptance are centrally focused on the role of evaluations, and non-judging (also called non-evaluation) is often considered a crucial facet of acceptance (Shallcross et al., 2010; Williams and Lynn, 2010).

Non-judging is also a construct that is featured prominently in the mindfulness literature, where it is construed as a core element of mindfulness (Kabat-Zinn, 1994; Bishop et al., 2004; Gethin, 2011). In this context, non-judging has been characteristically defined as “taking a non-evaluative stance toward thoughts and feelings” (Baer et al., 2008). Although rarely made explicit, it is generally implied that non-judging does not mean the absence of evaluations appearing in the mind. Instead, non-judging entails the capacity to release evaluations as well as the recognition that many evaluations are unnecessary (Kabat-Zinn, 2011).

Although some evaluations can cause emotional distress and distort our perception of reality (Sheeran et al., 2014), other evaluations may help us accurately interpret events and skillfully navigate our lives (Mrazek et al., 2017). As such, evaluations exist along a continuum ranging from counterproductive to helpful. Many conceptualizations of non-judging discourage both counterproductive and helpful evaluations in order to observe experiences in a relatively objective and unbiased manner. Nonetheless, other conceptualizations of non-judging emphasize foregoing only counterproductive evaluations (Robins and Chapman, 2004; Ford et al., 2018). Notably, the Five Factor Mindfulness Questionnaire includes questions that ask about evaluations in both ways (Baer et al., 2006). For example, “I make judgments about whether my thoughts are good or bad” (helpful or counterproductive) and “I believe some of my thoughts are abnormal or bad and I should not think that way” (only counterproductive). Accordingly, an important area for continued investigation is the relative merits of releasing all evaluations versus releasing only counterproductive evaluations.

Although many conceptualizations of acceptance emphasize the importance of foregoing all evaluation, many people find peace through evaluations like “this is part of God’s plan” and “this will make me stronger.” This illustrates that certain evaluations in the face of challenge can facilitate greater acceptance. Why then are these kinds of evaluations so rarely included in scientific frameworks of acceptance? One possibility is that they appear to be in direct contradiction with the aspect of non-judging. Yet it is worth noting that when non-judging is narrowly construed as foregoing counterproductive evaluations, the apparent incompatibility falls away. It is clearly possible to forego counterproductive evaluations while still making ones that facilitate greater acceptance.

## 5 Aspect four: non-attachment

In everyday speech, acceptance often refers to being okay with things as they are. Yet most of us have strong preferences for how we want things to be. We become attached to specific kinds of thoughts, feelings, and external circumstances—feeling averse to some while craving others. Furthermore, our emotional well-being also gets strongly tied to whether our preferences are met. Yet it is possible to release at least some of our personal preferences through non-attachment. Whereas non-judging involves letting go of evaluations, we refer to non-attachment as reducing the intensity of one’s personal preferences—including both desires and aversions. Although evaluations and preferences are deeply intertwined, they are also dissociable—as when someone evaluates broccoli as healthy but still feels an aversion to eating it.

In contemplative traditions, non-attachment is often cultivated through the recognition that inner experiences and worldly events are ultimately transient and unable to sustain a lasting peace and happiness (Kyabgon, 2014; Ricard, 2014). Additionally, many contemplatives point to an intrinsic peace within that is independent of circumstances but obscured by our habitual patterns of attachment (Gethin, 1998; Strong, 2015). Widely used self-report instruments intended to measure non-attachment have been inspired by these contemplative frameworks, and they include items like “As time goes on, I feel less and less of a need to be a certain way” (Sahdra et al., 2010; Whitehead et al., 2018). These instruments either implicitly or explicitly acknowledge that attachment is not an all-or-none phenomena. It exists along a continuum. With insight and practice, one can reduce their craving for positive experiences and reduce their aversion to negative experiences. Over time, this helps one develop a sense of impartiality, where one “approaches pleasant, unpleasant, and neutral experiences with equal interest” (Grabovac et al., 2011).

With this impartiality in mind, an important consideration is whether non-attachment can be differentiated from apathy and resignation. Apathy is commonly defined as a lack of interest or concern, while resignation is commonly defined as giving up in the face of something unfortunate. On the surface, it does initially appear paradoxical that someone could release all their personal preferences and yet still be motivated to engage with life. Yet contemplatives have argued that after releasing one’s personal preferences through non-attachment, one’s decisions and actions can be guided by wisdom, rationality, and concern for the well-being of others. Notably, non-attachment has indeed been associated with not only increased subjective well-being but also greater empathic concern and prosocial behavior (Sahdra et al., 2010, 2015). This suggests that non-attachment does not necessarily imply apathy or resignation, though continued research could further clarify what motivations persist or increase as one practices non-attachment.

Another important question is the relationship between non-attachment and equanimity. Equanimity is often described as emotional stability and mental composure in the face of provocative stimuli (Carmody et al., 2009) or as an even-minded mental state toward all experiences (Desbordes et al., 2015). Equanimity is also sometimes referred to as a facet of acceptance or even as being synonymous with acceptance. Furthermore, non-attachment can give rise to greater equanimity (Hadash et al., 2016; Eberth et al., 2019). Yet in our assessment, equanimity is a multifaceted outcome that could arise in a number of ways. Several aspects of acceptance described in this paper might increase one’s experience of equanimity. Even emotional suppression or certain psychoactive drugs might temporarily increase it as well. Accordingly, we view equanimity as a distinct construct from non-attachment whose relationship to the aspects of acceptance is deserving of continued investigation.

## 6 Dissociating aspects of acceptance

Some scientists describe acceptance as an umbrella term that encompasses a number of related constructs (see Lindsay and Creswell, 2017). It could be argued that all of these separate constructs—or at least a core subset of them—are so similar to one another or so inextricably linked that it is reasonable to describe and investigate them as essentially one thing (i.e., “acceptance”). However, our view is that while these various constructs may share similarities and even be mutually supportive of one another, there are nevertheless important conceptual and practical distinctions between them.

To illustrate this point, consider how often there are dissociations between the four aspects of acceptance discussed above. It is easy to identify instances where one might acknowledge something without “accepting” it in any other sense. For example, a person might acknowledge feeling anxious while still avoiding the feeling, evaluating the anxiety as problematic, and feeling a strong preference for the anxiety to disappear. If the person were to not only acknowledge their anxiety but also allow themselves to experience it fully, they could still evaluate the anxiety as problematic and hold a strong preference for it to be gone. Even a person who acknowledged, allowed, and refrained from judging their anxiety might still feel as though it would be better if they could get rid of it. To further illustrate these dissociations, Table 1 defines each of the four aspects of acceptance described above and gives an example of how that aspect could be present or absent in a common situation.

**Table 1:** Clarifying the aspects of acceptance through the example of feeling guilty.

Aspect	Definition	Aspect is present in example	Aspect is absent in example
Acknowledging	The recognition of something’s existence	I acknowledge that I feel guilty.	I do not feel guilty.
Allowing	Permitting something to exist in your experience	I am willing to let myself experience this guilt.	I am going to avoid this feeling of guilt.
Non-judging	Releasing evaluations about an experience	This feeling of guilt is not inherently a bad thing.	This feeling of guilt is pathetic.
Non-attachment	Reducing personal preferences, including both desires and aversions	I’m okay with feeling guilty.	I want this feeling of guilt to go away.

This table illustrates the aspects of acceptance by describing their presence or absence in a scenario where someone feels guilty.

## 7 The aspects of acceptance in acceptance and commitment therapy

The word acceptance is used in dozens of scientific and clinical frameworks, so it is far beyond the scope of this article to clarify how the four aspects of acceptance relate to each existing theory, measure, or therapeutic paradigm. Yet to further illustrate the aspects of acceptance, we will explore how they are incorporated into Acceptance and Commitment Therapy (ACT). Our hope is that even readers without any particular interest in ACT will find this section helpful in clarifying the four aspects of acceptance more generally.

ACT is a validated therapeutic modality that combines acceptance and mindfulness strategies with commitment and behavior-change strategies (Hayes, 2005). ACT includes six core processes, four of which are arguably related to acceptance as it is variously described in the scientific literature. Here we present each of these four processes—acceptance, being present, cognitive defusion, and self-as-context—and we describe how each process corresponds to the aspects of acceptance highlighted in this review.

Given variations in how each ACT process has been described in the scientific literature, we drew definitions from ACT-originator Steven Hayes’ most widely cited papers and books. Our intention is not to make any definitive claims about ACT. To the extent that others disagree with our characterization of the overlap between ACT processes and the aspects of acceptance, this may serve to reinforce our deeper point that there is considerable ambiguity about how the word acceptance is used in the scientific literature.

### 7.1 The ACT component of acceptance

In ACT, the word *acceptance* is used to describe “acknowledging and embracing the full range of experience” (Hayes et al., 2012). It is described as “an alternative to experiential avoidance” that is “supported by a willingness to make contact with distressing private experiences or situations, events, or interactions that will likely trigger them” (Hayes et al., 2006, 2012). Based on these definitions, acceptance within ACT refers to what we have described in this paper as *acknowledging* and *allowing*. This entails recognizing something’s existence rather than denying it (acknowledgment) and permitting it to exist in one’s current experience rather than avoiding it (allowing).

### 7.2 The ACT component of being present

The ACT process of being present refers to “ongoing non-judgmental contact with psychological and environmental events as they occur” (Hayes et al., 2012). ACT encourages people to use their thinking mind “more as a tool to note and describe events, not simply to predict and judge them” (Hayes, 2005). The ACT process of “being present” therefore has a strong correspondence to the non-judging aspect of acceptance described above.

Being present is also described as “choosing to pay attention to experiences here and now that are helpful or meaningful—and if they are not, then choosing to move on to other useful events in the now, rather than being caught in mindless attraction or revulsion” (Hayes, 2019). This characterization suggests that “being present” may reduce craving and aversion, so it may be related to the non-attachment aspect of acceptance. This characterization further suggests that within ACT, certain kinds of evaluations are encouraged (i.e., judging the helpfulness or meaning of experiences).

### 7.3 The ACT component of cognitive defusion

Within ACT, cognitive defusion is the process of “stepping back from the meaning of verbal processes and beginning to witness them from the point of view of an observer (Hayes et al., 2012).” It involves “seeing thoughts as they actually are—ongoing attempts at meaning-making—and then choosing to give them power only to the degree that they genuinely serve us” (Hayes, 2019). The intended result is to “decrease the believability of or attachment to private events rather than an immediate change in their frequency” (Hayes, 2005).

We suggest that cognitive defusion is not by itself one of the four aspects of acceptance described above. However, stepping back and decentering from inner experiences is intended to facilitate a stance of non-attachment. It may also make it easier to allow an experience to unfold without judgment. So while we would not describe cognitive defusion as an aspect of acceptance *per se*, it could help facilitate several of the aspects.

### 7.4 The ACT component of self-as-context

Within ACT, self-as-context refers to adopting the perspective that you are not the content of your thoughts and feelings. Instead, you are the consciousness that experiences those thoughts and feelings. Identifying as this “context” or “noticing self” involves “a sense of observing, witnessing, or purely being aware” (Hayes, 2019). By relating to experiences in this way, “one can be aware of one’s own flow of experiences without attachment to them or an investment in which particular experiences occur” (Hayes et al., 2006). Based on this definition, self-as-context is not synonymous with any of the four aspects of acceptance. However, it does have strong parallels with the aspect of non-attachment. By removing any personal identification with the content of experience, self-as-context allows a person to observe experiences with less craving or aversion.

### 7.5 Recap of the ACT components and the aspects of acceptance

As its name makes clear, ACT is a therapeutic modality that involves the practice of acceptance. Indeed, one of the six core components of ACT is called acceptance. Yet based on our assessment, several of the other ACT components also overlap with how the construct of acceptance is construed in other scientific frameworks.

The ACT component of acceptance refers to what we have described in this paper as acknowledging and allowing. The ACT component of being present refers primarily to non-judging. Finally, the ACT components of cognitive defusion and self-as-context are not themselves aspects of acceptance, though they can directly facilitate non-attachment. Attempting to identify these points of convergence was not straightforward, which highlights the ambiguity about how the word acceptance is used in the scientific literature.

## 8 Conclusion

In this review, we clarify four aspects of acceptance that are frequently referenced in the scientific literature—acknowledging, allowing, non-judging, and non-attachment. We hope this provides a vocabulary that will help researchers be more precise when communicating about their theories, frameworks, interventions, and findings. Arguably, it may be unrealistic for all scientists and clinicians to ever converge on a universally agreed upon definition of acceptance. Fortunately, even without that consensus, scientific understanding can still grow iteratively and achieve meaningful synthesis across studies if scientists and clinicians are careful to specify which constructs do and do not apply in their research. To this aim, we have provided a list of questions in Table 2 that we recommend researchers answer when publishing their work on acceptance.

**Table 2:** Concept worksheet for acceptance-related theories, measures, and interventions.

Aspect	Clarifying questions to report in manuscript or Supplementary materials
Acknowledging	Does it entail acknowledging something’s existence? If yes: Does this pertain to acknowledging all experiences or only some (e.g., “inner” vs. “outer” experiences, aversive vs. pleasant experiences?)
Allowing	Does it entail allowing something to exist in one’s current experience? If yes: Does this pertain to allowing all experiences or only some (e.g., inner vs. outer, aversive vs. pleasant)? Is there a specific orientation to experience (e.g., neutral, welcoming, embracing)? Does it include foregoing attempts to change experience? If yes, provide exceptions if applicable.
Non-judging	Does it entail foregoing evaluations/judgments? If yes: Does this pertain to all evaluations or only some (e.g., evaluations of inner vs. outer experiences, helpful vs. counterproductive evaluations, evaluations of aversive vs. pleasant experiences)?
Non-attachment	- Does it entail letting go of personal preferences (i.e., cravings and aversions)? If yes: Does this pertain to all preferences or only some (e.g., preferences for inner vs. outer experiences, aversive vs. pleasant experiences)?
Finally, describe any other aspects included and provide a definition of each.	

These questions are intended to clarify how an acceptance-related theory, measure, or intervention (hereafter “it”) relates to four distinct aspects of acceptance. We recommend including the most relevant details within your manuscript, as well as complete answers to these questions within your Supplementary materials.

One important consideration for future research not yet mentioned is whether the aspects of acceptance represent states or traits of an individual. Arguably, all four aspects of acceptance can be construed as both a state and a trait. For example, one can choose to release evaluations in any given moment (e.g., during therapy, meditation, or throughout daily activities). Over time, non-judging can also become an enduring trait that becomes one’s default stance toward experience. While this distinction between states and traits adds yet another layer of complexity, we hope the vocabulary presented here will help the scientific community navigate this and other challenges on its quest toward a coherent understanding of acceptance.

## Author contributions

MM: Writing – review & editing, Writing – original draft, Conceptualization. BD: Writing – review & editing, Writing – original draft, Conceptualization. JR: Writing – review & editing, Writing – original draft, Conceptualization. AP: Writing – review & editing, Writing – original draft, Conceptualization. NG: Writing – review & editing, Writing – original draft. TP: Writing – review & editing, Writing – original draft. BR: Writing – review & editing, Writing – original draft. AM: Conceptualization, Writing – review & editing, Writing – original draft.
